# Editorial: Carbon microelectrodes for neurochemical sensing

**DOI:** 10.3389/fbioe.2026.1799334

**Published:** 2026-02-06

**Authors:** Elisa Castagnola, Kendall H. Lee, Davide Ricci

**Affiliations:** 1 Department of Biomedical Engineering, Louisiana Tech University, Ruston, LA, United States; 2 Institute for Micromanufacturing, Louisiana Tech University, Ruston, LA, United States; 3 Department of Neurosurgery, LSU Health Shreveport, Shreveport, LA, United States; 4 Bioengineering Department, University of Pittsburgh, Pittsburgh, PA, United States; 5 Department of Neurologic Surgery, Myo Clinic, Rochester, MN, United States; 6 Department of Naval, Electrical, Electronic, and Telecommunications Engineering, Polytechnic School, University of Genoa, Genova, Italy

**Keywords:** carbon fiber microelectrodes, dopamine, fast scan cyclic voltammetry, glassy carbon, pyrolysis, serotonin

Carbon is widely regarded as an optimal material for electrochemical detection of neurotransmitters because it combines biocompatibility, high sensitivity to redox-active species, rapid electron-transfer kinetics, and excellent electrochemical stability, making it well suited for fast-scan cyclic voltammetry (FSCV) (Rafi and Zestos, 2021; Cao et al., 2019; Castagnola et al., 2010; Puthongkham and Venton, 2020; Roberts and Sombers, 2018). FSCV enables real-time detection of electroactive neurotransmitters such as dopamine (DA), serotonin (5-HT), and adenosine (AD) with sub-second resolution, revealing release and uptake dynamics relevant to neurological and psychiatric disorders (Huffman and Venton, 2009; Robinson et al., 2003; Venton and Cao, 2020; Swamy and Venton, 2007; Abdalla et al., 2017; At et al., 2015; Wood and Hashemi, 2013).

Carbon fiber microelectrodes (CFMEs) are considered the gold standard for FSCV: their ultrasmall diameters (5–10 µm), flexibility, and favorable electrochemical properties allow minimally invasive implantation and sensitive recording of transient neurochemical events with high spatiotemporal resolution (Devi et al., 2021; Bucher and Wightman, 2015; Alyamni et al.; Kozai et al., 2015). However, manual fabrication limits scalability and consistency, and chronic use is hindered by fouling and over-oxidation of the carbon surface, which progressively degrade mechanical and electrochemical performance (Kozai et al., 2015; Patel et al., 2020; Takmakov et al., 2010). Waveform strategies that extend the anodic limit can improve sensitivity and reduce fouling but accelerate oxidative etching, creating a trade-off between sensitivity and durability (Takmakov et al., 2010; Heien et al., 2003).

More recently, efforts to integrate neurochemical sensing into flexible neural probes for simultaneous electrophysiological recordings have led to the development of carbon-based microelectrode arrays (MEAs) incorporating glassy carbon (GC) (Castagnola et al., 2023; Castagnola et al., 2022; Castagnola et al., 2024), graphene (Li et al., 2022; Qi et al., 2025), or boron-doped diamond (BDD) (Fan et al., 2020; Castagnola et al., 2021; Castagnola et al., 2018) on flexible substrates. These devices support FSCV while providing reliable neural recordings. However, most designs still rely on metal interconnects, raising concerns about long-term reliability under chronic, high-frequency electrochemical cycling. “All”-GC-MEAs (Faul et al., 2024; Nimbalkar et al., 2018), which use GC for both electrodes and interconnects, mitigate these issues and exhibit excellent electrochemical stability, although further optimization is required to maintain scalability at very small feature sizes.

This Research Topic highlights advances in materials and fabrication strategies aimed at improving the chronic performance of CFMEs, enabling scalable batch manufacturing, and developing high-density GC-based MEAs capable of multimodal, multichannel recording with durable long-term stability.

Alyamni et al. present a comprehensive review of the design, fabrication, and applications of CFMEs and related carbon-based sensors, including GC and nanomaterial-enhanced electrodes. The review emphasizes progress in materials science and electrochemical methodologies to improve sensitivity, selectivity, and biocompatibility. It also details surface-modification strategies that mitigate fouling and enhance detection performance, as well as scalable manufacturing approaches—such as 3D printing and laser-induced graphene—that provide versatile, cost-effective solutions for fabricating carbon microelectrodes. Furthermore, the authors examine how electrode size, material choice, and coating chemistry influence tissue response and long-term stability. Clinical applications discussed include Parkinson’s disease, depression, and addiction research, underscoring the translational potential of these sensors. Overall, the review positions CFMEs as a rapidly advancing platform for neurochemical monitoring, while noting persistent challenges related to reproducibility, chronic stability, and scalable batch fabrication.

**FIGURE 1:**
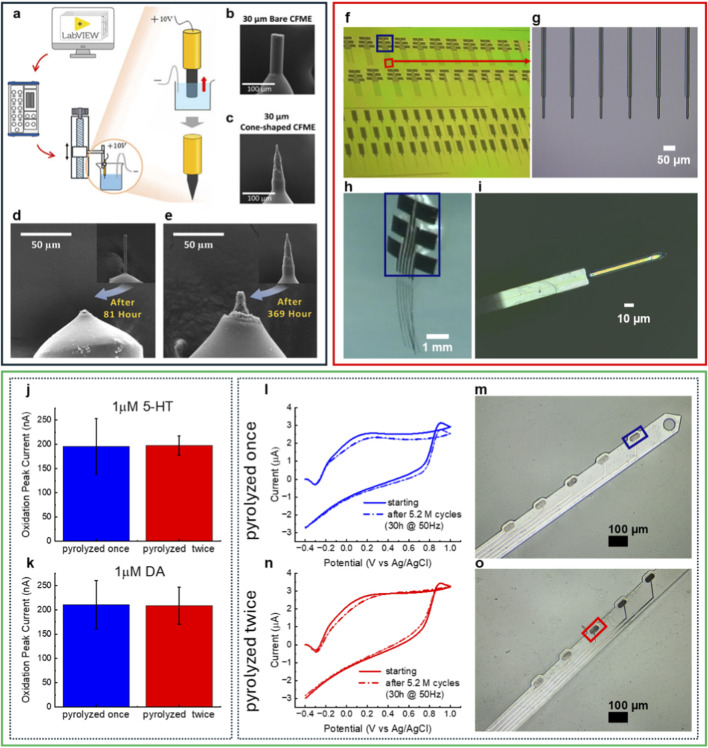
**(a–e)** adapted from (Kwon et al.). **(a)** Schematic of the homemade electrochemical etching system to create cone-shaped CFMEs. **(b,c)**; **(d–e)** Long term electrochemical durability of 7 μm and 30 µm cone-shaped CFMEs. SEM images of 7 μm **(d)** and 30 µm cone-shaped **(e)** CFMEs after continuous FSCV waveform application. **(f–i)** Adapted from (Siwakoti et al.). **(f)** Batch fabricated fGCF and fGCF arrays in a Si_3_N_4_ coated 4 in wafer before being released; **(g)** Optical picture of fGCF array with six GCFs at 170 μm each; **(h)** Optical picture of a released fGCF array; **(i)** Optical picture of fGCF (10 µm wide, 10 µm thick); **(j-o)** Adapted from (Sellen et al.). **(j–k)** Comparison of FSCV detection performance for 5-HT and DA using single- and double-pyrolyzed GC microelectrodes. **(j)** Comparison of 5-HT oxidation peak current measured using FSCV for the 2 GC conditions. **(k)** Comparison of DA oxidation peak current measured using FSCV for the 2 GC conditions. **(l)** Background current of a GC microelectrode obtained with a single pyrolysis step, measured before and after application of the FSCV waveform used for DA detection at 50 Hz for 30 consecutive hours. **(m)** Optical image of a GC-MEA shank with a magnified view of the stimulated electrodes (single pyrolysis, blue) *versus* non-stimulated electrodes. **(n)** Background current of a GC microelectrode obtained with double pyrolysis. **(o)** Optical image of a GC-MEA shank with a magnified view of the stimulated electrodes (double pyrolysis, red) *versus* non-stimulated electrodes.

Kwon et al. address a key challenge in maintaining stable FSCV performance during chronic recordings with conventional 7 µm CFMEs. To enhance mechanical robustness and long-term functionality, they developed 30 µm CFMEs and used electrochemical etching to create cone-shaped tips (Figures 1a–c). The *in vitro* and *in vivo* performance of 7 μm, 30 µm bare, and 30 µm cone-shaped CFMEs was compared using FSCV, alongside assessments of electrode longevity and brain-tissue response. Although 30 µm bare CFMEs demonstrated a 2.7-fold increase in sensitivity relative to 7 µm electrodes, *in vivo* DA detection decreased, likely due to greater insertion-induced tissue damage. Cone-shaped modification reversed this effect, producing a 3.7-fold increase in *in vivo* DA signal and reduced glial activation. Importantly, accelerated oxidative-etching tests under prolonged FSCV scanning revealed a 4.7-fold increase in lifespan compared with 7 µm CFMEs (Figures 1d,e). Collectively, these findings show that cone-shaped, larger-diameter CFMEs preserve low tissue damage and high *in vivo* sensitivity while extending lifespan compared with 7 µm microelectrodes, highlighting their suitability for chronic neurotransmitter monitoring.

Siwakoti et al. address the reproducibility and scalability limitations of manually fabricated CFMEs by introducing a double-etching microfabrication process for batch production of 10 μm × 10 µm full-GC fibers (fGCFs) and arrays composed entirely of homogeneous GC pyrolyzed from an SU-8 precursor (Figures 1f–i). Each fGCF incorporates a 2 µm low-stress silicon nitride bottom insulator and SU-8 top encapsulation, leaving a 150 μm GC segment exposed for sensing. Fabrication on 4-inch silicon wafers yielded up to 80 single or array devices per batch, demonstrating consistent scalability and reproducibility. Scanning electron microscopy (SEM) and energy-dispersive X-ray spectroscopy (EDS) confirmed uniform insulation and structural integrity, while electrochemical testing showed high conductivity, low impedance, and a wide potential window suitable for *in vitro* and *in vivo* sensing. Finite-element simulations guided optimization of the fGCF geometry for self-penetration up to 3 mm into the mouse striatum. *In vivo*, fGCFs detected both phasic DA via FSCV and tonic DA levels via square-wave voltammetry, while inducing minimal tissue damage. Their sensitivity, scalable fabrication, and self-supporting insertion capability make fGCFs promising minimally invasive sensors for multi-timescale DA monitoring.

Sellen et al. investigate next-generation “all”-GC-MEAs by examining the properties of GC both as a conductor and as a sensing material. The study evaluates how GC thickness, trace width, and trace length influence electrical behavior under single versus double pyrolysis, while simultaneously assessing how a second pyrolysis cycle affects GC performance as a neurochemical sensor. Sheet resistance, structural ordering (Raman/XRD), and FSCV sensing of DA and 5-HT were systematically assessed. Double pyrolysis caused ∼20% shrinkage and ∼88% higher sheet resistance, yet preserved structural integrity, electrochemical stability, and FSCV performance. Although GC shows higher resistance than ultrathin metal traces at very small widths, its resistance approaches metal-like values at 5–10 μm, supporting its feasibility for miniaturized interconnects. At the same time, the second pyrolysis cycle maintained sensing performance for both DA and 5-HT and preserved electrical stability under prolonged oxidative-etching FSCV tests (Figures 1j–o). Overall, this work provides key design guidance and shows that GC can reliably serve as both interconnect and sensing material, helping enable the next-generation of high-density “all”-GC-MEAs.

Together, this Research Topic highlights recent advances in carbon-based neurochemical interfaces, including progress in improving CFME chronic performance and in the batch fabrication of GC-based microsensors.
